# Lysostaphin and BMP-2 co-delivery reduces *S. aureus* infection and regenerates critical-sized segmental bone defects

**DOI:** 10.1126/sciadv.aaw1228

**Published:** 2019-05-17

**Authors:** Christopher T. Johnson, Mary Caitlin P. Sok, Karen E. Martin, Pranav P. Kalelkar, Jeremy D. Caplin, Edward A. Botchwey, Andrés J. García

**Affiliations:** 1 Wallace H. Coulter Department of Biomedical Engineering, Georgia Institute of Technology and Emory University, Atlanta, GA 30332, USA.; 2 Petit Institute for Bioengineering and Bioscience, Georgia Institute of Technology, Atlanta, GA 30332, USA.; 3 George W. Woodruff School of Mechanical Engineering, Georgia Institute of Technology, Atlanta, GA 30332, USA.

## Abstract

*Staphylococcus aureus* is the most common pathogen associated with bacterial infections in orthopedic procedures. Infections often lead to implant failure and subsequent removal, motivating the development of bifunctional materials that both promote repair and prevent failure due to infection. Lysostaphin is an anti-staphylococcal enzyme resulting in bacterial lysis and biofilm reduction. Lysostaphin use is limited by the lack of effective delivery methods to provide sustained, high doses of enzyme to infection sites. We engineered a BMP-2–loaded lysostaphin-delivering hydrogel that simultaneously prevents *S. aureus* infection and repairs nonhealing segmental bone defects in the murine radius. Lysostaphin-delivering hydrogels eradicated *S. aureus* infection and resulted in mechanically competent bone. Cytokine and immune cell profiling demonstrated that lysostaphin-delivering hydrogels restored the local inflammatory environment to that of a sterile injury. These results show that BMP-2–loaded lysostaphin-delivering hydrogel therapy effectively eliminates *S. aureus* infection while simultaneously regenerating functional bone resulting in defect healing.

## Teaser

Use of BMP-2 and lysostaphin-loaded hydrogels simultaneously clears *S. aureus* infection and repairs bone defects.

## INTRODUCTION

Effective treatment of infected segmental bone defects remains a considerable clinical challenge (*1*). Nonunion bone defects are a common clinical scenario accounting for more than 600,000 hospital cases per year totaling more than 5 billion U.S. dollars in costs (*2*). The current standard of care includes surgical placement of bone auto- and allografts to facilitate healing (*3*). However, these grafting procedures have failure rates as high as 13% (*4*). Furthermore, bacterial infection of bone grafts significantly increases implant failure rates, often leading to corrective surgery, including debridement of infected tissue, and significant morbidity to the patient (*5*). Up to 30% of nonunion injuries produce positive bacterial cultures, with staphylococcal species being the most common pathogen (*6*). Recently, the use of the Masquelet technique, a two-stage reconstruction protocol for segmental bone defects, has become popular in treating these infections (*7*). The first stage consists of extensive tissue debridement, limb stabilization followed by the placement of an antibiotic-eluting space maintainer, and ensuring soft tissue coverage and wound healing. The second stage of the procedure commences once the infection has been eradicated, often 4 to 8 weeks later. In this surgery, the cement spacer is removed, permanent fixation hardware is placed, and an autologous bone graft is harvested and deployed. The complex nature of this procedure motivates the development of materials that both promote bone regeneration and prevent failure due to infection using a single-stage surgical approach.

Lysostaphin is a metallo-endopeptidase produced by *Staphylococcus simulans* (*8*). This enzyme has antimicrobial activity specific against staphylococcal species. This specificity is provided by a targeting domain that binds the *Staphylococcus aureus* cell wall, and the antimicrobial activity is attributed to a catalytic domain that functions by cleaving the second and third glycine residues in the pentaglycine peptidoglycan cross bridges responsible for bacterial cell wall integrity, leading to cell lysis (*9*). The catalytic nature of lysostaphin makes its antimicrobial activity independent of the bacterial metabolic state, providing activity against sessile biofilm bacteria. This activity is in contrast to most small-molecule antibiotics that require metabolically active bacteria to be effective (*9*). Lysostaphin therapy prevents or reduces infection in several small-animal models (*9*, *10*). A clinical case report of systemic lysostaphin therapy in an unresponsive leukemia patient with methicillin-resistant *S. aureus* pneumonia, multiple abscesses, and cellulitis has been reported after failure of aggressive antibiotic therapy (*11*). The patient received intravenous infusion of 500 mg of lysostaphin. The reported side effects included flushing, mild hypotension, and tachycardia, which were effectively controlled. Blood, sputum, and abscess cultures were negative following lysostaphin administration until the patient’s death 3 days later, which was unrelated to the infection. Biomaterial carriers have been explored to increase lysostaphin stability and retention times at the site of administration, but retention times are still limited to a few hours (*12*, *13*). Biosynexus Corp. moved to commercialize lysostaphin cream to reduce intranasal *S. aureus* colonization. The phase 1/2 clinical trials demonstrated safety and efficacy, but upon completion, the current clinical standard, mupirocin, came off patent protection, making the further development of lysostaphin cream for intranasal infections economically insolvent (*14*). On the basis of these studies, there is significant potential for the use of lysostaphin to treat staphylococcal infections.

Because of the high failure rates of bone grafting to treat segmental defects, recombinant bone morphogenetic proteins (BMPs) have been under development for use in humans. BMP-2 has been approved by the U.S. Food and Drug Administration to facilitate bone formation in anterior lumbar interbody fusion, tibial fractures, and sinus augmentation procedures (*15*). However, for effective induction of bone formation, supraphysiological doses are delivered, which can result in several unintended side effects, such as ectopic bone formation, nerve damage, and significant inflammation (*16*). These limitations motivate the development of delivery carriers for controlled BMP-2 release to improve bone healing and reduce unintended side effects.

Hydrogels, water-swollen cross-linked polymer networks, can be engineered to deliver a wide array of protein therapeutics. Our laboratory has engineered four-arm poly(ethylene glycol)-maleimide (PEG-4MAL) hydrogels for the delivery of both protein and cell-based therapeutics (*10*, *17*, *18*). This synthetic delivery platform provides a well-defined hydrogel mesh structure, mild reaction conditions that are minimally toxic to cells, precise stoichiometric incorporation of biomolecules and cell-adhesive ligands, and control over the polymerization kinetics for injectable or precast gel delivery. In addition, the hydrogel small degradation products have low toxicity and are excreted through the urine. We previously engineered PEG-4MAL hydrogels functionalized with the collagen-mimetic cell-adhesive peptide GFOGER and loaded with BMP-2 to treat critical-sized murine radial segmental bone defects (*18*). These BMP-2–loaded hydrogels regenerated bone of superior quality compared to the clinical standard of a BMP-2–loaded collagen sponge. We recently showed that PEG-4MAL hydrogels delivering lysostaphin to murine femoral fractures infected with *S. aureus* eradicated the infection, allowing complete fracture healing (*10*).

We hypothesized that BMP-2 and lysostaphin co-delivery would eradicate *S. aureus* infection in bone defects, allowing bone regeneration and functional healing. We synthesized protease-degradable PEG-4MAL hydrogels functionalized with GFOGER and loaded with BMP-2 and lysostaphin, and delivered them to mouse radial segmental defects to treat *S. aureus* infection. We then evaluated their efficacy through a combination of bacterial viability counts, microcomputed tomography (μCT) imaging, histology, and mechanical testing. We also assessed the immunological response to the therapy through a combination of cytokine and immune cell profiling.

## RESULTS

### Lysostaphin-delivering hydrogels eliminate *S. aureus* infection in a mouse radial segmental defect

**Fig. 1:**
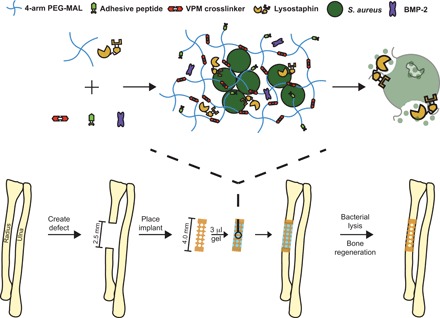
Lysostaphin and BMP-2 co-delivery to a critical-sized segmental bone defect. A 2.5-mm segment of the radius is removed to create a critical-sized bone defect that does not spontaneously heal. A PEG-4MAL hydrogel functionalized with the adhesive ligand GFOGER and loaded with lysostaphin and BMP-2 is synthesized with *S. aureus*. Lysostaphin enzymatically creates holes in the bacterial cell wall, leading to lysis. These infected hydrogel scaffolds are loaded into a 4-mm polyimide sleeve and placed over the ends of the defect. Co-delivery of BMP-2 and lysostaphin results in infection clearance followed by subsequent defect regeneration.

We set out to engineer a bone reparative hydrogel that eliminates *S. aureus* infection through the delivery of lysostaphin. We used a murine radial segmental defect model, in which a 2.5-mm nonhealing segmental bone defect was created in the mouse radius (Fig. 1). The hydrogel containing bacteria was precast inside of a 4.0-mm polyimide sleeve and placed over each end of the radius within the defect. Bone regeneration was then assessed longitudinally using μCT imaging. We synthesized lysostaphin- and BMP-2–delivering hydrogels in a one-pot reaction by mixing the protease-degradable cross-linking peptide GCRDVPMSMRGGDRCG (VPM) and the cell-adhesive peptide GGYGGP(GPP)_5_GFOGER(GPP)_5_GPC (GFOGER), which was covalently incorporated into the network via a peptide terminal cysteine residue, with a four-arm maleimide–terminated PEG (PEG-4MAL) macromer (Fig. 1). Both lysostaphin and BMP-2 were physically entrapped within the hydrogel mesh structure, as neither protein has free thiols available for covalent tethering onto the hydrogel network. To induce the infection component to the model, we added *S. aureus* to the hydrogel. Viability studies demonstrated that, in the brief time that hydrogels were maintained before implantation (<4 hours), no significant loss in live bacteria occurred in hydrogels containing lysostaphin (fig. S1). Consistency in delivered bacteria dose among experiments was assessed by assaying extra bacteria containing implants and comparing the bacterial counts to previous experiments.

First, we characterized the release of lysostaphin and BMP-2 from the hydrogels by incorporating fluorescently labeled lysostaphin and BMP-2 protein and measuring the fluorescence in supernatant with and without collagenase. The results showed that lysostaphin and BMP-2 diffuse from the hydrogel network and that the addition of collagenase, which proteolytically degrades the network, increases the release rate of the enzyme (fig. S2). This result shows that lysostaphin freely diffuses through the gel, providing for killing of bacteria within and surrounding the material, and that, upon exposure to protease to degrade the hydrogel network, the release rate is increased.

We then tested the ability of lysostaphin-delivering hydrogels to eliminate *S. aureus* infection using the mouse segmental defect infection model. *S. aureus* Xen29, a luminescent strain derived from an abdominal wound infection [American Type Culture Collection (ATCC) 12600], was chosen for this initial study. *S. aureus–*infected and sterile hydrogels, not containing BMP-2, with and without lysostaphin were delivered to the murine radial bone defect, and microbiologic analysis was performed after 7 days to assess viable bacteria. Complete eradication of *S. aureus* was observed in the infection group treated with lysostaphin-delivering hydrogel (fig. S3A), demonstrating that lysostaphin-delivering hydrogels successfully prevent infection in radial segmental defects. Histologic analyses via hematoxylin & eosin (H&E) and safranin-O and fast green revealed no differences between sterile hydrogels and Xen29-containing hydrogels treated with lysostaphin, whereas untreated hydrogels demonstrated a robust inflammatory response (fig. S3B).

To further support the idea that lysostaphin-delivering hydrogels eliminate bone infections, we implanted hydrogels infected with UAMS-1, a clinical isolate from a case of pediatric osteomyelitis and heavy biofilm former (*19*). UAMS-1–infected hydrogels containing 50 ng of BMP-2, with and without lysostaphin, and sterile controls were implanted in the murine bone defect, and microbiological analysis was performed 8 weeks after implantation. Lysostaphin-delivering hydrogel therapy eliminates infection at 8 weeks, whereas untreated gels are grossly infected (fig. S4).

### Lysostaphin-delivering hydrogels regenerate bone in infected radial defects

**Fig. 2:**
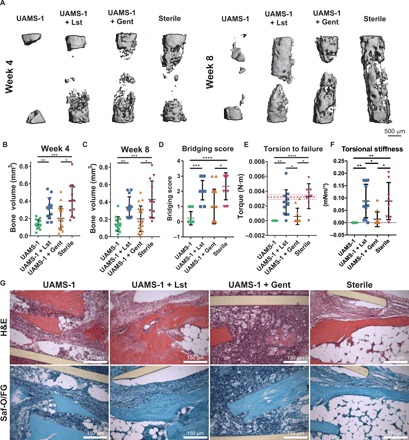
UAMS-1 infection defects treated with BMP-2–loaded lysostaphin-delivering hydrogels significantly improve bone repair. Representative μCT reconstructions at 4 and 8 weeks. (**A**) Quantification of bone volume from μCT imaging at 4 (**B**) and 8 (**C**) weeks after implantation. Kruskal-Wallis test with Dunn’s multiple comparisons test. Means ± SD. *n* = 12 to 18 per group. *P < 0.05, **P < 0.01, ***P < 0.001. (**D**) Defect bridging was assessed semiquantitatively using the following scale: 0, no bone formation; 1, less than half of the defect; 2, greater than half of the defect; 3, defect bridged. Kruskal-Wallis with Dunn’s multiple comparisons test. *n* = 12 to 18 per group. *P < 0.05, ***P < 0.001, ****P < 0.0001. (**E**) Functional healing was assessed using torsion to failure testing. The average torsion to failure values for healthy mouse radii are plotted as horizontal red lines (0.0032 ± 0.0003 N·m), as reported by Shekaran *et al*. (**F**) Torsional stiffness was then calculated for all samples. Means ± SD. *n* = 8 to 11 per group. One-way analysis of variance (ANOVA) with Tukey’s post hoc test for torsion to failure and Kruskal-Wallis with Dunn’s multiple comparisons test for torsional stiffness. (**G**) Mouse radii were sectioned and stained with H&E and safranin-O/fast green (Saf-O/FG). One sample was randomly selected and prepared per experimental group. Representative images are displayed.

The development of a bacterial biofilm can significantly complicate the treatment of segmental bone defects, resulting in multiple surgeries. Initially, the wound is debrided to remove all infected and necrotic tissue, and long-term systemic antibiotics are administered to clear any remaining infection and subsequent surgeries are then required to repair the bone defect (*7*). To test whether BMP-2–loaded lysostaphin-delivering hydrogels can prevent biofilm infection and regenerate bone, we focused on the *S. aureus* strain UAMS-1. Segmental bone defects were created, and hydrogel scaffolds loaded with 100 ng of BMP-2 and 1 U of lysostaphin were deployed. Longitudinal bone formation was monitored using μCT imaging at 4 and 8 weeks. At the end of the 8-week study, mechanical testing and histologic analyses were performed on the mouse forelimbs. The experimental groups included infected hydrogels with and without lysostaphin and sterile hydrogels without lysostaphin. We also included a group of infection-only hydrogels containing 100 ng of BMP-2 that were dipped in gentamicin (10 mg/ml) (*20*) to simulate the current therapeutic standard of locally delivered antibiotic-doped implants. Representative μCT reconstructions at 4 and 8 weeks after implantation (Fig. 2A) showed no new bone formation in the untreated infection group over the entire 8-week course. Both the lysostaphin-delivering and sterile hydrogel groups exhibited new bone formation inside the defect over the course of the 8-week experiment. Hydrogels that were treated with gentamicin also showed new bone formation. However, some samples in the gentamicin group were completely devoid of new bone, suggesting that not all of the infections were cleared, which was also supported by abscesses visible at the time of necropsy. Quantification of bone volume from the reconstructed μCT images at 4 weeks (Fig. 2B) showed significantly increased bone volume in the lysostaphin-delivering hydrogel and sterile control groups compared to the untreated infections. There was no difference in bone volume between the lysostaphin-delivering hydrogel group and the sterile control. Local treatment with gentamicin showed no improvement in bone formation compared to the infection control. Similar results were observed at 8 weeks (Fig. 2C). We also quantified the extent of defect bridging by scoring the 8-week μCT reconstructions: 0, no bone formation; 1, less than half of the defect; 2, greater than half of the defect; 3, defect bridged (Fig. 2D) (*18*). This analysis indicated that the sterile and lysostaphin-delivering hydrogels had higher defect bridging scores compared to the untreated control. Implants treated with gentamicin were not different from the infection implants. Together, these results show that BMP-2–loaded lysostaphin-delivering hydrogels significantly improve bone regeneration compared to untreated infections and regenerate equal amounts of bone as sterile implants.

We next assessed the mechanical integrity of the regenerated bone with torsion to failure testing (Fig. 2E). The results show significantly increased mechanical properties for both infections treated with lysostaphin/BMP-2–delivering hydrogel and the sterile control group. There was no difference in the mechanical integrity of the regenerated bone between these two groups. Infected hydrogels treated with gentamicin were not different from the infected controls, and the mechanical properties for the repair tissue for these groups were significantly lower than those for BMP-2/lysostaphin–delivering hydrogels and sterile controls. In addition, the sterile control group torsion to failure value was similar to that of normal intact bone [3.2 ± 0.3 mN·m, as reported by Shekaran *et al*. (*18*)], and the average torsion values measured for infections treated with lysostaphin-delivering hydrogels were not statistically different from naïve bone. Torsional stiffness was also calculated, and the results mirror the trends of the torque-to-failure testing (Fig. 2F). Histologic analysis at 8 weeks after implantation revealed the presence of inflammatory cell infiltrate and no new bone formation within the defect for the infection-only and gentamicin-treated samples, indicating a persistent infection (Fig. 2G). Notably, infections treated with a lysostaphin-delivering hydrogel had similar morphology to that of the sterile control. Bone was present within the defect site, and a marrow cavity was formed. Collectively, these results demonstrate that BMP-2–loaded lysostaphin-delivering hydrogels eliminate infection and drive defect repair, leading to functional bone regeneration.

### Lysostaphin-delivering hydrogels restore the native inflammatory environment

As a protein that does not occur naturally in the human body, lysostaphin could trigger an inflammatory reaction and negatively affect bone formation. Furthermore, lysostaphin functions by directly lysing bacteria, which, in itself, could cause a significant inflammatory response through the release of bacterial by-products. We performed liver enzyme testing at 1 week after operation to test whether BMP-2–loaded lysostaphin-delivering hydrogel therapy resulted in liver toxicity. The experimental groups included lysostaphin-delivering hydrogels with and without UAMS-1 infection containing 100 ng of BMP-2. Liver function was assessed by measuring total protein, albumin, aspartate aminotransferase, alanine aminotransferase, and alkaline phosphatase. Overall, no differences were observed among groups, and the results were largely within normal limits (fig. S5). We also screened for anti-lysostaphin antibody generation using a dot blot assay. Anti-lysostaphin antibodies were present in 1 of 20 animals before surgery. Four weeks following implantation, three of seven sterile lysostaphin-delivering hydrogel–treated animals were positive for anti-lysostaphin antibodies. One of six animals in the infection-only control group (no lysostaphin) and one of seven in the infections treated with lysostaphin-delivering hydrogels were antibody positive, with no differences in the number of animals that converted from an antibody-negative status before implantation to antibody-positive status at any time point (*P* > 0.05, χ^2^ test). These data indicate that lysostaphin-delivering hydrogels do not result in liver toxicity but may lead to the formation of anti-lysostaphin antibodies.

**Fig. 3:**
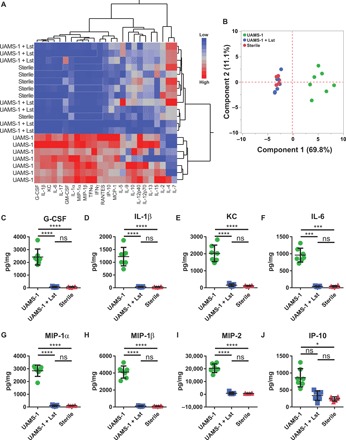
BMP-2–loaded lysostaphin-delivering hydrogels restore the local inflammatory environment to a regenerative state 1 week after implantation. Segmental defects were created, hydrogel scaffolds infected with UAMS-1 with or without lysostaphin as well as sterile gels were implanted, and the inflammatory response was assessed using a multiplexed cytokine array assay 1 week later. (**A**) Hierarchical cluster analysis of cytokine profiles using the Ward method. (**B**) PCA of the array data. (**C** to **J**) Cytokines with statistically different tissue levels as determined using two-way ANOVA with Bonferroni correction for multiple comparisons. Means ± SD. *n* = 6 to 7 per group. *P < 0.05, ***P < 0.001, ****P < 0.0001. ns, not significant.

**Fig. 4:**
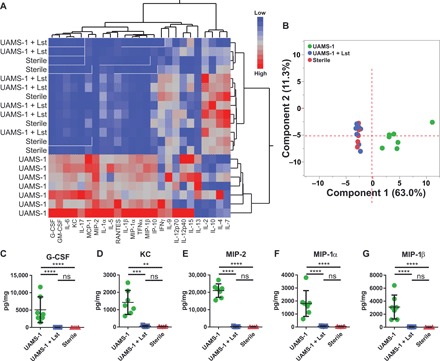
Cytokine profile of BMP-2–loaded lysostaphin-delivering hydrogels 4 weeks post-operatively. Segmental defects were created, hydrogel scaffolds infected with UAMS-1 with or without lysostaphin as well as sterile gels were implanted, and the inflammatory response was assessed using a multiplexed cytokine array assay 4 weeks later. (**A**) Hierarchical cluster analysis of cytokine profiles using the Ward method. (**B**) PCA of the array data. (**C** to **G**) Cytokines with statistically different tissue levels as determined using two-way ANOVA with Bonferroni correction for multiple comparisons. Means ± SD. *n* = 6 to 7 per group. *P < 0.05, ***P < 0.001, ****P < 0.0001.

We characterized the local cytokine milieu generated in response to BMP-2–loaded lysostaphin-delivering hydrogel therapy. Hydrogels were placed in the bone defects, and 1 and 4 weeks after implantation, the implant tube and surrounding tissue were isolated and analyzed using a multiplexed cytokine array assay. The experimental groups included UAMS-1 bacteria-containing hydrogels with and without lysostaphin and lysostaphin-free sterile controls. All implants contained 100 ng of BMP-2. At the 1-week time point, hierarchical cluster analysis revealed clear segregation of the untreated infection group from the sterile control and lysostaphin-delivering hydrogel group (Fig. 3A). Notably, no separation between the sterile and lysostaphin hydrogel–treated groups was apparent, suggesting that the infection had been cleared and the local inflammatory environment had been restored to that of a sterile wound environment. To compare the cytokine levels among experimental groups, we performed principal components analysis (PCA) across all cytokines examined (Fig. 3B). The results showed principal component 1 being responsible for 69.8% of the variability, with clear separation between the infection control group and infections treated with lysostaphin-delivering hydrogels. The sterile and lysostaphin treatment groups were indistinguishable from one another. We then analyzed individual cytokine levels. Several cytokines were significantly elevated in the infection-only control group, including granulocyte colony-stimulating factor (G-CSF), interleukin-1β (IL-1β), keratinocyte chemoattractant (KC), IL-6, macrophage inflammatory protein-1α (MIP-1α), MIP-1β, MIP-2, and interferon (IFN)–inducible protein 10 (IP-10) (Fig. 3, C to J). These cytokines are primarily associated with the acute inflammatory response and are largely responsible for inflammatory cell recruitment (*21*). No significant differences were detected between infections treated with lysostaphin-delivering hydrogels and the sterile controls. Notably, these same trends continued at the 4-week time point. Hierarchical cluster analysis separated the infection-only group from both the sterile and lysostaphin-delivering hydrogel–treated groups, which were indistinguishable from each other (Fig. 4A). PCA also revealed similar global cytokine levels. Both sterile and lysostaphin-delivering hydrogel–treated mice do not form independent clusters, while both formations can be discriminated from the infection-only controls by principal component 1 (Fig. 4B). This result is further supported by individual cytokine analyses, which showed significantly elevated levels of G-CSF, KC, MIP-2, MIP-1α, and MIP-1β in the UAMS-1 infection mice compared to sterile and lysostaphin hydrogel–treated infected groups (Fig. 4, C to G). Again, these cytokines are primarily associated with inflammatory cell recruitment, showing a continued immune response to the persistent infection. Fewer differences in individual cytokine expression levels were detected at the 4-week time point compared to 1 week, which may be attributed to the local inflammatory profile shifting from an acute to a more chronic state (*22*). No differences were detected between the sterile and lysostaphin-delivering hydrogel–treated mice, indicating that lysostaphin-delivering hydrogels return the local inflammatory environment to that of a pro-healing state.

To further characterize the host response to BMP-2–loaded lysostaphin-delivering hydrogels to treat *S. aureus* infection, we profiled the inflammatory cell populations at the defect site via flow cytometry. Segmental bone defects were created, and lysostaphin-delivering hydrogels loaded with 100 ng of BMP-2 were implanted. The experimental groups included hydrogels with and without lysostaphin that were implanted with and without infection. At 1 and 4 weeks following implantation, mice were euthanized, tissue was isolated, and single-cell suspensions were stained for inflammatory cell markers (table S1). The gating strategy used to identify individual cell types is outlined in fig. S6. Both absolute counts and cell percentages were determined on live cells.

**Fig. 5:**
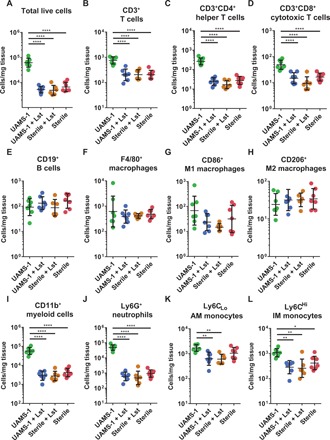
Total number of inflammatory cells at 1 week after implantation of BMP-2–loaded lysostaphin-delivering hydrogels. One week following segmental defect creation and implant placement, mice were euthanized, the implant and surrounding tissue were recovered, and flow cytometry was performed to enumerate the total number of inflammatory cells present. (**A**) Total cells, (**B**) CD3^+^ T cells, (**C**) CD3^+^CD4^+^ helper T cells, (**D**) CD3^+^CD8^+^ cytotoxic T cells, (**E**) CD19^+^ B cells, (**F**) F4/80^+^ macrophages, (**G**) CD86^+^ M1 macrophages, (**H**) CD206^+^ M2 macrophages, (**I**) CD11b^+^ myeloid cells, (**J**) Ly6G^+^ neutrophils, (**K**) Ly6C_low_ AM monocytes, and (**L**) Ly6C^high^ IM monocytes were enumerated. Data were log-transformed, and ordinary one-way ANOVA with Tukey’s post hoc test was used. Means ± SD. *n* = 6 to 7 per group. *P < 0.05, **P < 0.01, ***P < 0.001, ****P < 0.0001. IM, inflammatory monocyte; AM, anti-inflammatory monocyte.

At 1 week after implantation, the infection-only control defects had significantly increased numbers of total cells (Fig. 5A), CD3^+^ T cells (Fig. 5B), CD3^+^CD4^+^ helper T cells (Fig. 5C), CD3^+^CD8^+^ cytotoxic T cells (Fig. 5D), CD11b^+^ myeloid cells (Fig. 5I), Ly6G^+^ neutrophils (Fig. 5J), and Ly6C^high^ classical inflammatory monocytes (Fig. 5L) compared to lysostaphin-delivering hydrogel–treated infections and both sterile control groups. Significantly higher levels of alternative/anti-inflammatory monocytes were observed in the infection-only group compared to infections treated with lysostaphin-delivering hydrogels and sterile lysostaphin-delivering hydrogels (Fig. 5K). Whereas the infection-only group showed a significant increase in total cells (fig. S7), the myeloid cell population in the infection-only group is dominated by neutrophils. The higher numbers of inflammatory cells observed in the infection-only group were accompanied by visible tissue abscesses present at necropsy. No differences in cell frequency at the defect site were observed among the lysostaphin-delivering hydrogel–treated infections and both sterile control groups (Fig. 5, A to L), demonstrating that lysostaphin-delivering hydrogel formulations do not produce hyper-inflammatory conditions. There were no differences in the total number of B cells (Fig. 5E) or macrophages (Fig. 5, F to H) between any of the experimental groups, although the total number of cells was increased in the infection-only group, suggesting that these are tissue-resident macrophages.

**Fig. 6:**
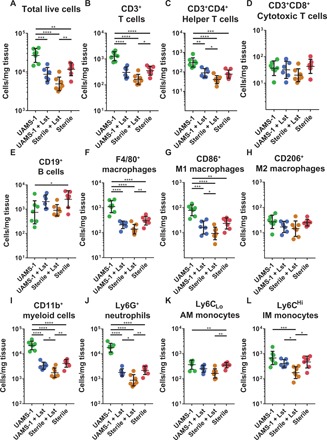
Total number of inflammatory cells at 4 weeks after implantation of BMP-2–loaded lysostaphin-delivering hydrogels. Four weeks following segmental defect creation and implant placement, mice were euthanized, the implant and surrounding tissue were recovered, and flow cytometry was performed to enumerate the total number of inflammatory cells present. (**A**) Total cells, (**B**) CD3^+^ T cells, (**C**) CD3^+^CD4^+^ helper T cells, (**D**) CD3^+^CD8^+^ cytotoxic T cells, (**E**) CD19^+^ B cells, (**F**) F4/80^+^ macrophages, (**G**) CD86^+^ M1 macrophages, (**H**) CD206^+^ M2 macrophages, (**I**) CD11b^+^ myeloid cells, (**J**) Ly6G^+^ neutrophils, (**K**) Ly6C_low_ AM monocytes, and (**L**) Ly6C^high^ IM monocytes were enumerated. Data were log-transformed, and ordinary one-way ANOVA with Tukey’s post hoc test was used. Means ± SD. *n* = 6 to 7 per group. *P < 0.05, **P < 0.01, ***P < 0.001, ****P < 0.0001.

At 4 weeks after implantation, increased numbers of total cells (Fig. 6A), CD3^+^ T cells (Fig. 6B), CD3^+^CD4^+^ helper T cells (Fig. 6C), F4/80 macrophages (Fig. 6F), M1 macrophages (Fig. 6G), and neutrophils (Fig. 6J) were present in the infection-only group compared to infections treated with lysostaphin-delivering hydrogels and both sterile control groups. The increased number of myeloid-derived cells is primarily composed of neutrophils for the infection-only group in response to infection. No differences were observed in the number of CD3^+^CD4^+^ cytotoxic T cells (Fig. 6D) or M2 macrophages (Fig. 6H) among any of the groups. While differences in absolute quantitates in several cell types were observed among several cell types in the infections treated with lysostaphin-delivering hydrogels and the sterile control groups (Fig. 6, A to C, F, G, and J to L), no differences were observed in the percent of parent analysis (fig. S8). Moreover, lysostaphin-delivering hydrogel–treated infections and both sterile control groups exhibited an increased percentage of M2 macrophages, suggesting that a pro-regenerative phenotype is present (fig. S8). The variability in absolute cell quantification is likely attributable to the differences between the total cells recovered among groups at later time points.

## DISCUSSION

Nonhealing segmental bone defects often require the placement of a bone auto- or allograft, or the use of BMP-2, to induce osteogenesis and promote defect healing. However, these injuries carry rates of infection reported as high as 30%, making it critically important that infection be prevented and/or eliminated, to facilitate healing (*6*, *23*). We engineered a synthetic hydrogel that both induces bone regeneration and eliminates infection through the co-delivery of BMP-2 and lysostaphin, respectively. We showed that BMP-2–loaded lysostaphin-delivering hydrogels eradicate infection and regenerate bone with similar mechanical properties to those of intact mouse radii. Liver function testing revealed no signs of systemic toxicity, although anti-lysostaphin antibodies were generated in some subjects. At the infection site, lysostaphin-delivering hydrogels returned the inflammatory environment to that of an uninfected defect, as measured by both cytokine secretion and immune cell profiling. These results support the development of BMP-2–loaded lysostaphin-delivering hydrogels to prevent *S. aureus* infection and promote segmental bone defect regeneration.

Infection prevention is critical to the success of segmental bone defect repair (*24*, *25*). Injuries resulting in segmental bone defects carry a disproportionately high infection rate compared to other orthopedic injuries, often due to the complexity of the injury, such as exposed bone fragments (*26*). Treatment requires extensive surgical debridement to remove necrotic bone and tissue, followed by the placement of an antibiotic-impregnated spacer. A second procedure is performed at a later time, once the infection has cleared, to remove the spacer and place the bone graft (*7*). Even with proper surgical intervention, infection remains one of the primary causes of nonunion (*6*). Interventions that fight infection while also being osteoinductive have the potential to significantly reduce the incidence of nonunion, leading to improved patient outcomes. Antimicrobial prophylaxis is a critical component to treating segmental bone defects (*6*, *23*). Current strategies have centered on adding broad-spectrum antimicrobial therapy to an osteoinductive scaffold (*27*). Alternatives to broad-spectrum antimicrobials, such as silver (*28*) and d-amino acids (*29*, *30*), have also been shown to be effective at reducing infection and promoting bone repair.

Lysostaphin provides potent bacteriospecific antimicrobial activity. This targeted approach ensures that only the pathogen of interest is eliminated, but may not be an ideal approach to prevent infection, when the target is often unknown and broad-spectrum agents are desirable (*31*). Furthermore, it is possible that prevention of a single pathogen may allow other opportunistic pathogens to colonize the injury site, such as with a less common gram-negative pathogen-like *Pseudomonas aeruginosa*. However, in orthopedic cases, roughly two-thirds of all infections are staphylococcal in nature (*32*), providing lysostaphin therapy significant prophylactic coverage using this targeted approach. Our strategy could be expanded to include other species-specific antimicrobial agents, such as bacteriophage lysins (*33*) or antimicrobial peptides (*34*), thereby enhancing its translational potential as a prophylactic agent (*31*).

The synthetic nature of our PEG-4MAL hydrogels functionalized with the GFOGER peptide and loaded with recombinant BMP-2 protein has several advantages over traditional bone grafting procedures. Synthetic scaffolds do not require graft procurement, which will eliminate donor site morbidity. Removing this aspect of the procedure could reduce the opportunity for intraoperative infection by shortening the procedure times (*35*, *36*). In addition, the “off-the-shelf” nature of this synthetic delivery vehicle is scalable to any defect size. Lysostaphin-delivering hydrogels are inherently adhesive and can be delivered via injection, allowing the material to coat and fill any gaps within the injury for easy administration to orthopedic injuries that may be very anatomically complex and heterogeneous (*10*). However, further studies will need to be performed in larger animal models to confirm that larger BMP-2–loaded lysostaphin-delivering hydrogels can regenerate bone in larger defects.

Bacterial infection triggers a robust inflammatory response to clear the pathogen from the body. Our histologic analysis indicated that *S. aureus* infection resulted in the development of significant leukocytic infiltrate. Both the cytokine and immune cell profiling further supported this conclusion. At 1 week after infection, several cytokines responsible for immune cell recruitment were significantly up-regulated in infected-only control defects, including G-CSF, MIP-1α, MIP-1β, MIP-2, KC, IP-10, IL-6, and IL-1β. These cytokines are central to neutrophil recruitment (*37*, *38*), and the flow cytometry results support this observation. This result shows a robust innate immune response to the infection, and our data show that lysostaphin-delivering hydrogels are able to either prevent this response to infection from occurring or restore the inflammatory environment to normal levels through the elimination of bacteria. Notably, no differences in the cellular response to the material were observed between sterile empty hydrogels and sterile lysostaphin-delivering hydrogels, supporting the conclusion that lysostaphin does not, in itself, trigger a significant inflammatory response.

In terms of the adaptive immune response, we observed significantly elevated levels of T cells including both helper and cytotoxic T cells in the untreated infection animals. The percentage of cytotoxic T cells remained constant in the infection-only group compared to both sterile controls, but both the number and proportion of helper T cells were significantly elevated. Helper T cells are required for B cell antibody affinity maturation and class switching (*39*). They are also involved in the recruitment of neutrophils and macrophages from bone marrow, thereby promoting phagocytosis (*40*), consistent with the argument that this increase in helper T cells is evidence of a typical adaptive immune response to infection. Other groups have characterized the inflammatory cells present in bone infection sites for a limited number of cell types, such as myeloid-derived suppressor cells (MDSCs) (*41*–*43*) and B cells (*44*) as well as T cell subsets located in the draining lymph node and spleen (*45*). Vidlak and Kielian (*46*) have also reported MDSC, monocyte, macrophage, and CD3^+^ T cell compositions in the context of prosthetic joint infections. To our knowledge, the present study is the most comprehensive report of immune cell subtypes for bone-associated *S. aureus* infections.

In these studies, the hydrogel and the bacteria are delivered together, proving a model of infection prevention (prophylaxis). In many clinical cases, injuries occur, and then intervention follows sometime later, allowing bacteria to begin colonizing the injury before treatment. This motivates further development of a delayed treatment model (*47*). Follow-up studies should consider placing bacteria at the defect site before placing the hydrogel construct, as well as treating established bone defect infections with lysostaphin/BMP-2–loaded hydrogels in a subsequent surgery.

In conclusion, we have engineered a lysostaphin- and BMP-2–delivering hydrogel that simultaneously eradicates *S. aureus* infection and repairs critical-sized mouse radial segmental bone defects with bone of similar quality to that of healthy tissue. This bacteriospecific strategy is targeted against *Staphylococcus* species. Future studies could investigate the incorporation of antimicrobial agents that could expand the activity spectrum. These hydrogels show no signs of systemic or local toxicity, supporting further investigation using higher-order animal models.

## MATERIALS AND METHODS

### Bacterial strains and culture

*S. aureus* strains UAMS-1 (ATCC 49230) and Xen29 (Perkin-Elmer) were grown at 37°C on TSA (trypticase soy agar) or Luria-Burtani agar supplemented with kanamycin (50 μg/ml), respectively.

### Lysostaphin and BMP-2 co-delivering hydrogel synthesis

Hydrogels were synthesized using four-arm 20-kDa PEG-4MAL macromer (Lysan Bio) functionalized with the collagen-mimetic peptide GGYGGP(GPP)_5_GFOGER(GPP)_5_GPC (GFOGER) (New England Peptide), lysostaphin (AMBI), *S. aureus* UAMS-1, or Xen29 and then cross-linked using the cysteine-flanked protease-degradable peptide GCRDVPMSMRGGDRCG (VPM). All components were suspended in 100 mM MES–buffered phosphate-buffered saline (PBS) at a pH of 5.5 to 6.0. Single colonies of UAMS-1 or Xen29 were picked from agar plates and suspended in PBS to an optical density of 0.20 measured at 600 nm using a benchtop spectrophotometer (MicroScan Turbidity Meter, Siemens). The bacterial suspension was diluted 100-fold for UAMS-1 studies and 10-fold for Xen29 studies. BMP-2 (R&D Systems) was prepared at 333 ng/ml in 4 mM HCl. For hydrogels with lysostaphin, the enzyme was added to PEG-MAL. Hydrogels were synthesized by mixing 4:2:2:1:1 parts PEG-4MAL, GFOGER, VPM, BMP-2, and bacteria, followed by injection into a polyimide tube with 300-μm laser machined holes (MicroLumen). The final hydrogel composition was 4.0% (w/v) 20-kDa PEG-4MAL, 1.0 mM GFOGER, 412 ± 85 colony-forming units (CFU)/gel UAMS-1, 100 ng of BMP-2, with or without 1 U of lysostaphin. Hydrogels were polymerized at 37°C and 5% CO_2_ for 15 min, then swollen in PBS cut into 4-mm segments, and kept in PBS until implantation.

### Murine radial segmental defect infection model

All live animal experiments were performed in accordance with the Institutional Animal Care and Use Committee at Georgia Institute of Technology under veterinary supervision. Ten- to 12-week-old male C57/B6 mice (The Jackson Laboratory) were anesthetized via isoflurane inhalation. Depilatory cream was used to remove fur from the right forelimb. The limb was surface-disinfected by applying 70% isopropyl alcohol followed by chlorohexidine solution. Slow release buprenorphine (1 mg/kg) was injected intraperitoneally before surgery as an analgesic. A 1-cm incision was made on the right forelimb over the radius, followed by blunt dissection of the radius. A 2.5-mm section of the radius was then excised using a custom-made double-bladed bone cutting device. A 4-mm polyimide implant tube containing the hydrogel was then fitted over each end of the radius. For mice receiving local gentamicin therapy, infected UAMS-1–containing implants were dipped in gentamicin (10 mg/ml) (*20*), followed by dipping in 0.9% (w/v) sodium chloride before implantation. The wound was sutured closed, and an x-ray image (MX-20 Radiography System, Faxitron) was taken of the radius to confirm appropriate implant placement. Mice were placed under warming lamp and monitored until ambulatory.

### Recovery of bacteria from tissue samples

Mice were euthanized via CO_2_ inhalation, the right forelimb was sterilized with 70% isopropyl alcohol, and the skin was removed. The implant tube, surrounding tissue, and bone were removed, weighed, and kept on ice. Tissue samples from the mouse forelimb were homogenized using bead beating tubes (1.4-mm zirconium beads, OPS Diagnostics) in combination with a FastPrep-24 system (MP Biomedicals) set to 6 m/s for a total of five successive runs, 40 s in duration. Liver samples were homogenized for 10 s using a LabGEN 7 (Cole-Palmer) tissue homogenizer in 12 mm × 75 mm sterile tubes. Single-cell bacterial suspensions were then prepared by a series of sonication and vortexing steps (10-min sonication, 30-s vortex, 5-min sonication, 30-s vortex, 30-s sonication, 30-s vortex). These single-cell suspensions were then serially diluted in PBS, plated on agar plates, incubated overnight at 37°C, and enumerated. Bacterial counts were normalized to tissue weight and reported as CFU/mg or CFU/implant if tissue weights were not recorded.

### μCT and bone volume quantification

μCT of mouse radii was performed as previously described with minor modifications (*18*, *48*). Animals were anesthetized via isoflurane inhalation, and a 3.2-mm-long section centered over the radial defect was imaged using a vivaCT system (Scano Medical) with the following imaging parameters: intensity, 145 μA; energy, 55 kVp; integration time, 200 ms; resolution, 15 μm. Contours of the radius within the implant tube were drawn on each two-dimensional section, and a Gaussian filter was applied (sigma, 1; support, 1; threshold, 540 mg of hydroxyapatite per cubic centimeter) to quantify bone volume.

### Mechanical testing of radii

Mechanical testing was performed as previously described (*18*). Briefly, mice were euthanized via CO_2_ inhalation, and the right forelimb was dissected, wrapped in saline-soaked gauze, and frozen at −20°C until the time of analysis. Samples were thawed under running deionized water, and the radius and ulna and surrounding tissue were removed from the forelimb. The ulna was then cut at its midpoint using a scalpel blade to ensure that the mechanical integrity of the radius was evaluated. Samples were potted in Woods metal containing blocks. Torsion to failure testing was performed using a Bose ElectroForce ELF 3200 system in conjunction with a 0.07 N·m torque sensor (Transducer Techniques) by applying a constant rotation of 3°/s. The maximum recorded torque value was reported. Torsional stiffness was determined by plotting torque against degrees rotation and calculating the slope using linear regression of the linear region of the measurement.

### Dot blot for anti-lysostaphin antibody generation

Pre-exposure blood samples were collected at the time of surgery via cheek bleed. Post-exposure blood samples were collected 4 weeks after surgery by cardiac puncture after CO_2_ euthanasia. Blood samples were clotted and centrifuged, and serum was collected and stored at −80°C until analysis. The dot blot assay was performed using the vacuum-driven Manifold I Spot-Blot System (Schleicher & Schuell). The nitrocellulose membrane was coated with lysostaphin (100 μg/ml; AMBI), blocked, and washed, and then 10,000-fold diluted serum samples were exposed to the membrane. A mouse anti-lysostaphin polyclonal immunoglobulin G (IgG; Antibody Research Corporation) was used as a positive control. Anti-lysostaphin antibodies were detected using an Alexa Fluor 488–conjugated polyclonal goat anti-mouse IgG antibody (Abcam). A Typhoon FLA 9500 gel imager (GE Healthcare) was used to image the membrane. ImageQuant (GE Healthcare) was used to quantify blot intensity. Positive results were determined to be five times the average intensity of serum samples from animals that were not exposed to lysostaphin.

### Liver function analysis

Mice were euthanized by CO_2_ inhalation, and blood was taken via cardiac puncture. Serum was separated, and samples were sent for blood chemistry testing at Antech Diagnostics.

### Histology of tissue samples

Mice were euthanized via CO_2_ asphyxiation, and the right forelimb was dissected, fixed in 10% neutral-buffered formalin, and decalcified in formic acid. Samples were then processed and embedded in paraffin. Sections (5 μm) of the radius and implant tube were cut and stained with either H&E, safranin-O and fast green, or gram stain using standard methods. Color images of the tissue sections were taken with a Nikon Eclipse E600 microscope using a Plan Fluor 20× objective (Nikon), MicroPublisher 5.0 RTV (QImaging) color camera, and QCapture software (QImaging).

### In vivo flow cytometry analysis

Flow cytometry analysis of tissue samples was performed as previously described (*49*). Briefly, mice were euthanized via CO_2_ asphyxiation, and the right forelimb was dissected. The implant tube and surrounding tissue were removed, weighed, and digested in collagenase type 1A (1 mg/ml, Sigma) at 37°C for 45 min. Following digestion, samples were separated using a cell strainer to form a single-cell suspension. The single-cell suspensions were stained for analysis using standard methods. The samples were analyzed on a FACSAria III flow cytometer (BD Biosciences). The antibodies used for cell staining were as follows: Alexa Fluor 488–conjugated anti-CD206 (BioLegend), BV421-conjugated anti-CD19 (BioLegend), BV605-conjugated anti-CD4 (BioLegend), BV785 anti-CD8a (BioLegend), phycoerythrin (PE)/Cy7–conjugated anti-CD3ε (BioLegend), BV510-conjugated anti-Ly6C (BioLegend), allophycocyanin (APC)–conjugated anti-F4/80 (BioLegend), APC/Cy7-conjugated anti-Ly6G (BioLegend), and PE-conjugated anti-CD86 (BioLegend). Live/dead staining was performed using the Zombie Red Fixable Viability Kit per the manufacturer’s instructions (BioLegend). Precision counting beads (BioLegend) were used to report absolute cell numbers.

### In vivo cytokine array analysis

Following euthanasia via CO_2_ inhalation, the right forelimb was dissected and the implant tube and surrounding tissue were removed for processing. The samples were placed in radioimmunoprecipitation assay buffer, minced, and sonicated. The homogenate was centrifuged at 10,000*g* for 5 min, and the supernatant was filtered using a 0.45-μm spin filter, snap-frozen in liquid nitrogen, and stored at −80°C until the time of analysis. A MilliPlex 25-plex mouse cytokine array kit (Millipore Sigma) was used to quantify tissue concentrations of G-CSF, GM-CSF (granulocyte-macrophage CSF), IFN-γ, IL-1α, IL-1β, IL-2, IL-4, IL-5, IL-6, IL-7, IL-9, IL-10, IL-12p40, IL-12p70, IL-13, IL-15, IL-17, IP-10, KC, MCP-1 (monocyte chemoattractant protein-1), MIP-1α, MIP-1β, MIP-2, RANTES, and TNF-α (tumor necrosis factor–α) per the manufacturer’s instructions. Results were read using a Luminex system (Luminex Corporation) and normalized to total protein content of the sample measured with a bicinchoninic acid assay kit (Pierce by Thermo Fisher Scientific). Samples below or above the detection limit of the assay were reported as the minimum or maximum value, respectively.

### Lysostaphin and BMP-2 release from PEG-4MAL hydrogels

The release profile of lysostaphin and BMP-2 from the PEG hydrogels was evaluated by fluorescently labeling the amine groups on the proteins, followed by monitoring the emission of fluorescence from the hydrogels containing these proteins. Briefly, in separate reactions, solutions of lysostaphin (AMBI) and BMP-2 (R&D Systems) were mixed with a solution of AF 594 *N*-hydroxysuccinimide ester dye (Fluoroprobes) (10× by moles as compared to the moles of the protein) in bicarbonate buffer (pH 8.3) for 1.5 hours. The reaction mixture was concentrated using centrifugal filters (Amicon) to remove any unreacted dye [molecular weight cutoff (MWCO) of 10,000 for lysostaphin reaction and MWCO of 3000 for BMP]. The concentrate was further purified using ÄKTA pure 25 (GE Healthcare) in combination with a HiPrep 16/60 Sephacryl S-100 HR size exclusion column with PBS as an elution buffer at 4°C. The eluents collected from the fast protein liquid chromatography column were concentrated and used in the preparation of the gels [4% (w/v) 20-kDa PEG-4MAL, 1 mM GFOGER, VPM crosslinker]. Each gel contained one fluorescently labeled protein and one unlabeled protein. To study the rate of diffusion, the gels were placed in PBS buffer at 37°C and the emission of fluorescence from the supernatant was measured using a Synergy H4 (BioTek) plate reader (585/626-nm excitation/emission wavelength) at various time points. For the protease-triggered release study, the gels were shaken in PBS containing collagenase type 1 enzyme (10 U/ml) (Worthington), and the supernatant was analyzed via fluorescence spectroscopy at various time points. Data were normalized to PEG-4MAL/lysostaphin/BMP mixtures suspended in the corresponding swelling supernatant of the respective hydrogel conditions.

### Statistics

All data were plotted as individual data points (biological replicates), with a line indicating the mean and the error bars representing the SD of the mean. A *P* value of less than 0.05 was deemed statistically significant. Statistical comparisons between two groups were made with Student’s *t* test. Multivariate parametric data were analyzed using analysis of variance (ANOVA) with Tukey’s post hoc test, and nonparametric data were analyzed using the Kruskal-Wallis test with Dunn’s multiple comparisons test. One-phase association curves were fit to the diffusion data, and extra sum of squares *F* test was performed to determine whether the data sets are different. A two-way ANOVA with a Bonferroni correction was used to identify statistically significant cytokines. A χ^2^ test was used to assess differences in the frequency of anti-lysostaphin antibody generation. All calculations were performed using Prism (GraphPad). Hierarchal cluster analysis was performed on the cytokine data using JMP Pro 13.

## Supplementary Material

http://advances.sciencemag.org/cgi/content/full/5/5/eaaw1228/DC1

Download PDF

## SUPPLEMENTARY MATERIALS

Supplementary material for this article is available at http://advances.sciencemag.org/cgi/content/full/5/5/eaaw1228/DC1 Supplementary Methods Fig. S1. Co-encapsulation of UAMS-1 and lysostaphin in the hydrogel system does not affect bacterial viability. Fig. S2. BMP-2–loaded lysostaphin-delivering hydrogels exhibit diffusion-mediated and protease-triggered release. Fig. S3. Lysostaphin-delivering hydrogels eliminate infection at 1 week. Fig. S4. Lysostaphin-delivering hydrogels eliminate infection at 8 weeks. Fig. S5. BMP-2–loaded lysostaphin-delivering hydrogels do not show signs of systemic toxicity. Fig. S6. Gating strategy for inflammatory cell profiling analysis. Fig. S7. Percent of parent inflammatory cells at 1 week after implantation of BMP-2–loaded lysostaphin-delivering hydrogels. Fig. S8. Percent of parent inflammatory cells at 4 weeks after implantation of BMP-2–loaded lysostaphin-delivering hydrogels. Table S1. Immune cell profiling antibody characteristics.
