# Supplementary material for: Lysostaphin and BMP-2 co-delivery reduces S. aureus infection and regenerates critical-sized segmental bone defects
Source: Sci Adv. 2019 May 17;5(5):eaaw1228. doi: 10.1126/sciadv.aaw1228 (PMC6524983; doi:10.1126/sciadv.aaw1228)
Supplement: http://advances.sciencemag.org/cgi/content/full/5/5/eaaw1228/DC1 [file supp_5_5_eaaw1228__index.html]

Science Advances | Science Advances

## Supplementary Materials

**This PDF file includes:**

- Supplementary Methods
- Fig. S1. Co-encapsulation of UAMS-1 and lysostaphin in the hydrogel system does not affect bacterial viability.
- Fig. S2. BMP-2–loaded lysostaphin-delivering hydrogels exhibit diffusion-mediated and protease-triggered release.
- Fig. S3. Lysostaphin-delivering hydrogels eliminate infection at 1 week.
- Fig. S4. Lysostaphin-delivering hydrogels eliminate infection at 8 weeks.
- Fig. S5. BMP-2–loaded lysostaphin-delivering hydrogels do not show signs of systemic toxicity.
- Fig. S6. Gating strategy for inflammatory cell profiling analysis.
- Fig. S7. Percent of parent inflammatory cells at 1 week after implantation of BMP-2–loaded lysostaphin-delivering hydrogels.
- Fig. S8. Percent of parent inflammatory cells at 4 weeks after implantation of BMP-2–loaded lysostaphin-delivering hydrogels.
- Table S1. Immune cell profiling antibody characteristics.

Download PDF

**Files in this Data Supplement:**

- Adobe PDF - aaw1228\_SM.pdf
