# Supplementary material for: Lysostaphin and BMP-2 co-delivery reduces S. aureus infection and regenerates critical-sized segmental bone defects
Source: Sci Adv. 2019 May 17;5(5):eaaw1228. doi: 10.1126/sciadv.aaw1228 (PMC6524983; doi:10.1126/sciadv.aaw1228)
Supplement: Download PDF [file aaw1228_SM.pdf]

Science Advances
AAAS

advances.sciencemag.org/cgi/content/full/5/5/eaaw1228/DC1

Supplementary Materials for

Lysostaphin and BMP-2 co-delivery reduces *S. aureus* infection and regenerates critical-sized segmental bone defects

Christopher T. Johnson, Mary Caitlin P. Sok, Karen E. Martin, Pranav P. Kalelkar, Jeremy D. Caplin, Edward A. Botchwey, Andrés J. García*

*Corresponding author. Email: andres.garcia@me.gatech.edu

Published 17 May 2019, *Sci. Adv.* **5**, eaaw1228 (2019)
DOI: 10.1126/sciadv.aaw1228

This PDF file includes:

Supplementary Methods
Fig. S1. Co-encapsulation of UAMS-1 and lysostaphin in the hydrogel system does not affect bacterial viability.
Fig. S2. BMP-2–loaded lysostaphin-delivering hydrogels exhibit diffusion-mediated and protease-triggered release.
Fig. S3. Lysostaphin-delivering hydrogels eliminate infection at 1 week.
Fig. S4. Lysostaphin-delivering hydrogels eliminate infection at 8 weeks.
Fig. S5. BMP-2–loaded lysostaphin-delivering hydrogels do not show signs of systemic toxicity.
Fig. S6. Gating strategy for inflammatory cell profiling analysis.
Fig. S7. Percent of parent inflammatory cells at 1 week after implantation of BMP-2–loaded lysostaphin-delivering hydrogels.
Fig. S8. Percent of parent inflammatory cells at 4 weeks after implantation of BMP-2–loaded lysostaphin-delivering hydrogels.
Table S1. Immune cell profiling antibody characteristics.

Supplementary Materials

Supplementary Methods

Live/Dead staining analysis

Briefly, hydrogels (20 µL) were synthesized using a bacterial concentration 50 times higher than the *in vivo* studies to achieve a concentration high enough for practical microscopic analysis. Hydrogels synthesized with and without lysostaphin were incubated in PBS at room temperature for 4 hours to simulate conditions prior to implantation. Afterwards, live/dead staining was performed using BacLight Bacterial Viability Kit L7007 (Life Technologies Corporation), in which 1 µL of Component A/Component B mixture was added to the gel and incubated for 15 minutes before analysis. Confocal microscopy was performed using a Nikon C2 Eclipse Ti inverted microscope using a 10X objective and a 488 nm laser. SYTO 9 fluorescence was detected using a 525/50 nm filter cube and propidium iodide was detected using a 595/50 nm filter cube. Images were analyzed using Fiji software. Cell counting was performed on each individual filter for all conditions, in which percent live bacteria was determined by the number of counts on the live filter divided by the total counts.

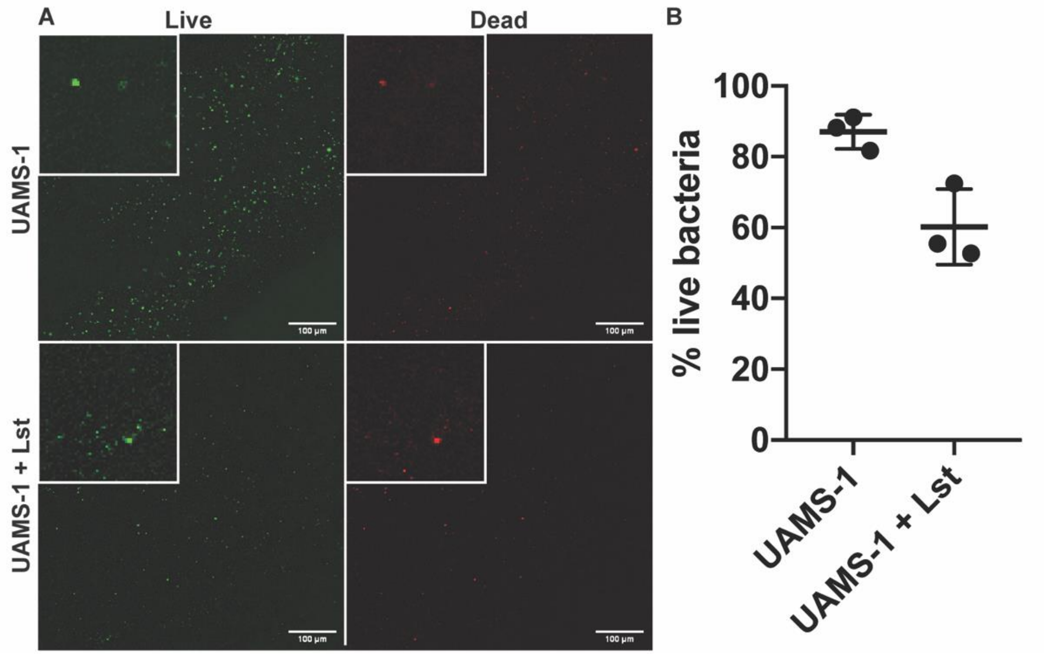

Fig. S1. Co-encapsulation of UAMS-1 and lysostaphin in the hydrogel system does not affect bacterial viability. (A) Representative images of live (green) and dead (red) bacteria encapsulated in hydrogels with and without lysostaphin 4 hours after hydrogel synthesis. (B) Percentage of live bacteria as determined by image analysis. Mann-Whitney test. Mean ± SD. N=3 per group. No differences were detected between groups.

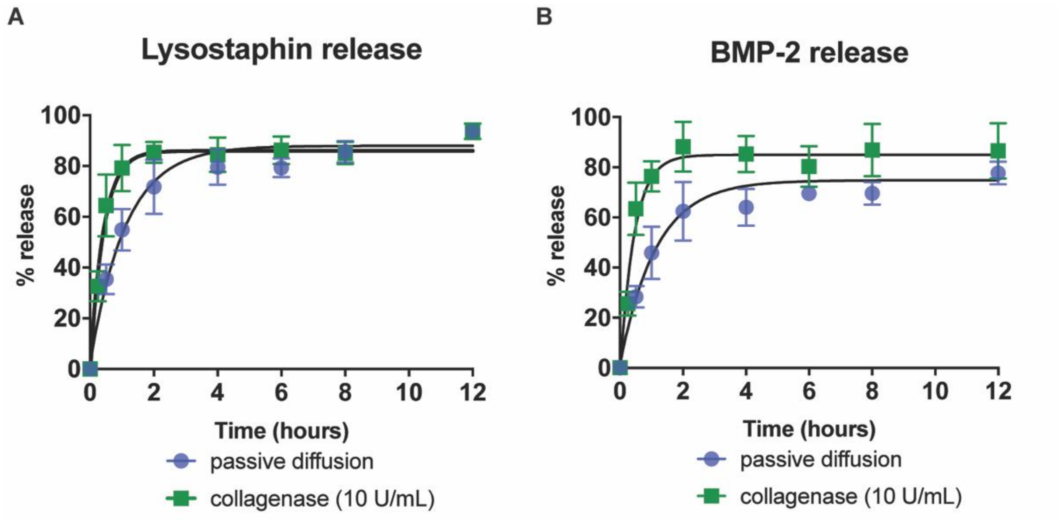

Fig. S2. BMP-2–loaded lysostaphin-delivering hydrogels exhibit diffusion-mediated and protease-triggered release. (A) Lysostaphin release from BMP-2 loaded, lysostaphin delivering hydrogels with and without collagenase. (B) BMP-2 release from BMP-2 loaded lysostaphin delivering hydrogels with and without collagenase. One-phase association fit using extra sum of squares F test to compare all K values are different. Means ± SD. *N*=5 per group.

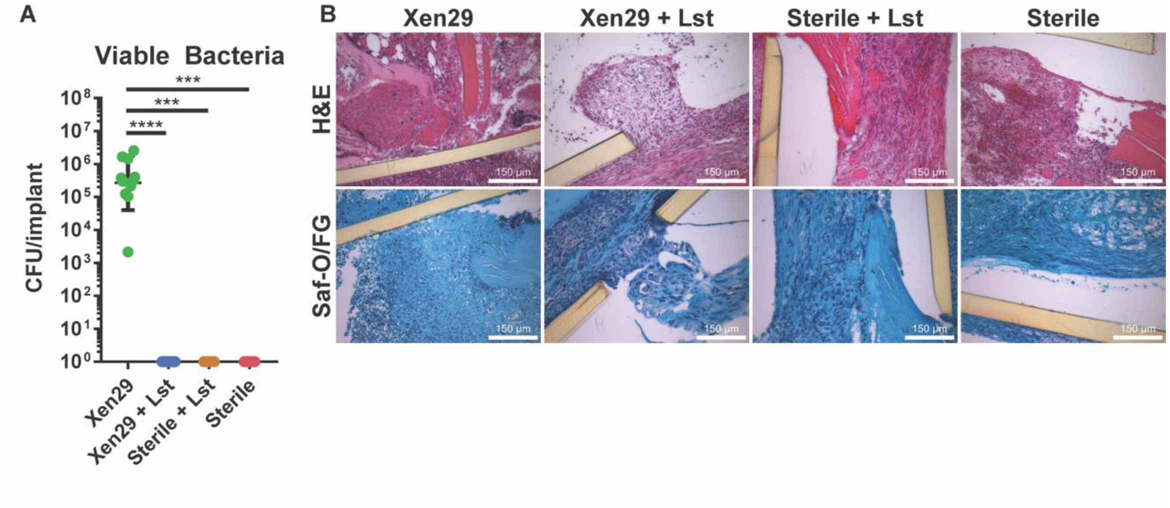

Fig. S3. Lysostaphin-delivering hydrogels eliminate infection at 1 week. (A) Viable bacteria recovered from the implant and surrounding tissue one week after implantation. (B) Histologic images of tissue sections stained with hematoxylin & eosin (H&E) and safranin-O/fast green (Saf-O/FG). Kruskal-Wallis test with Dunn's multiple comparaisons test. Means ± SD. N=5-11 per group. *** $P < 0.001$; **** $P < 0.0001$.

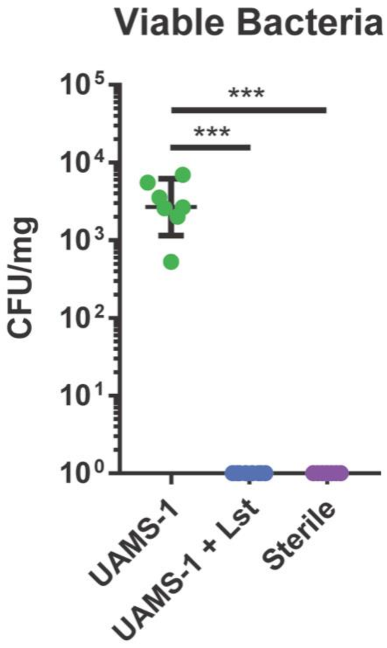

Fig. S4. Lysostaphin-delivering hydrogels eliminate infection at 8 weeks. Viable bacteria recovered from UAMS-1 infected radial defects 8 weeks post-implantation. Kruskal-Wallis test with Dunn's multiple comparisons test. Means ± SD. N=7-8 per group. *** $P < 0.001$.

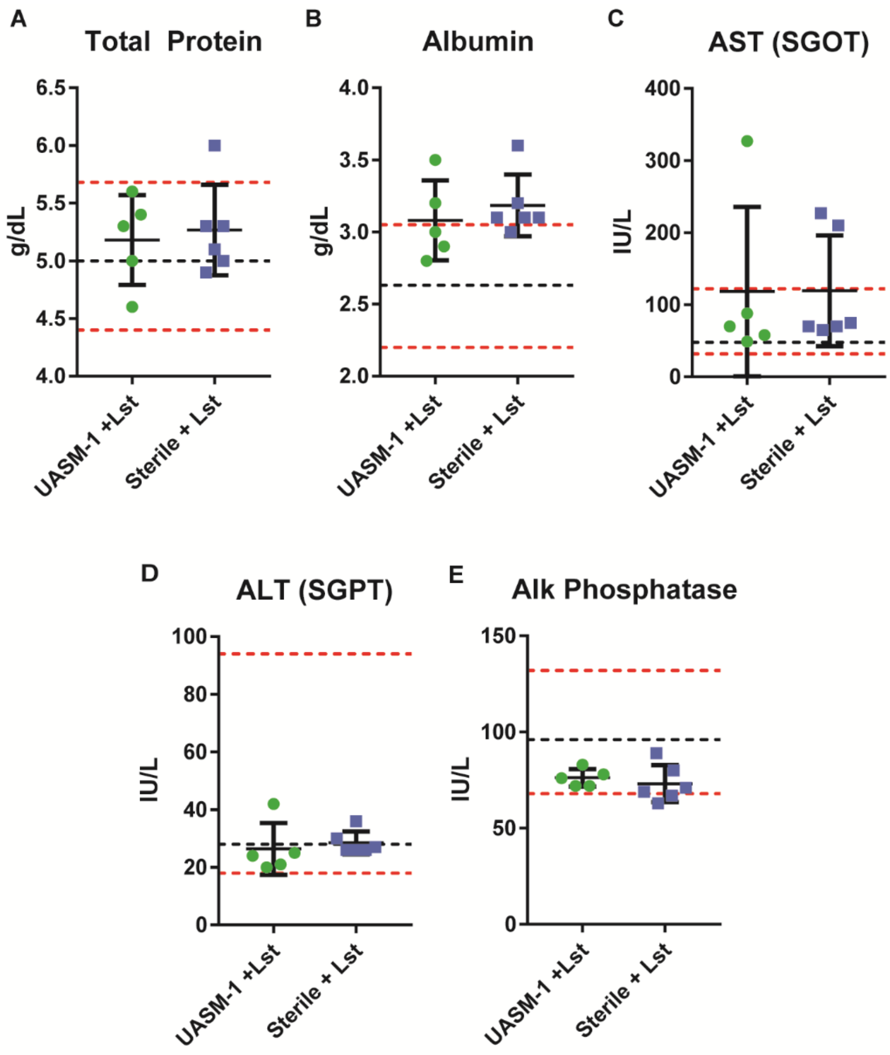

Fig. S5. BMP-2–loaded lysostaphin-delivering hydrogels do not show signs of systemic toxicity. Segmental defects were created and lysostaphin-delivering hydrogels loaded were BMP-2 with and without infection were implanted. Systemic toxicity was assessed at 1 week with liver function testing. Serum samples were tested for total protein (A), albumin (B), aspartate aminotransferase (AST) (C), alanine aminotransferase (ALT) (D), and alkaline phosphatase (Alk phosphatase) (E) levels. Means ± SD. *N*=5-6 per group. One-way ANOVA with Tukey's post-hoc test. No differences were detected between groups.

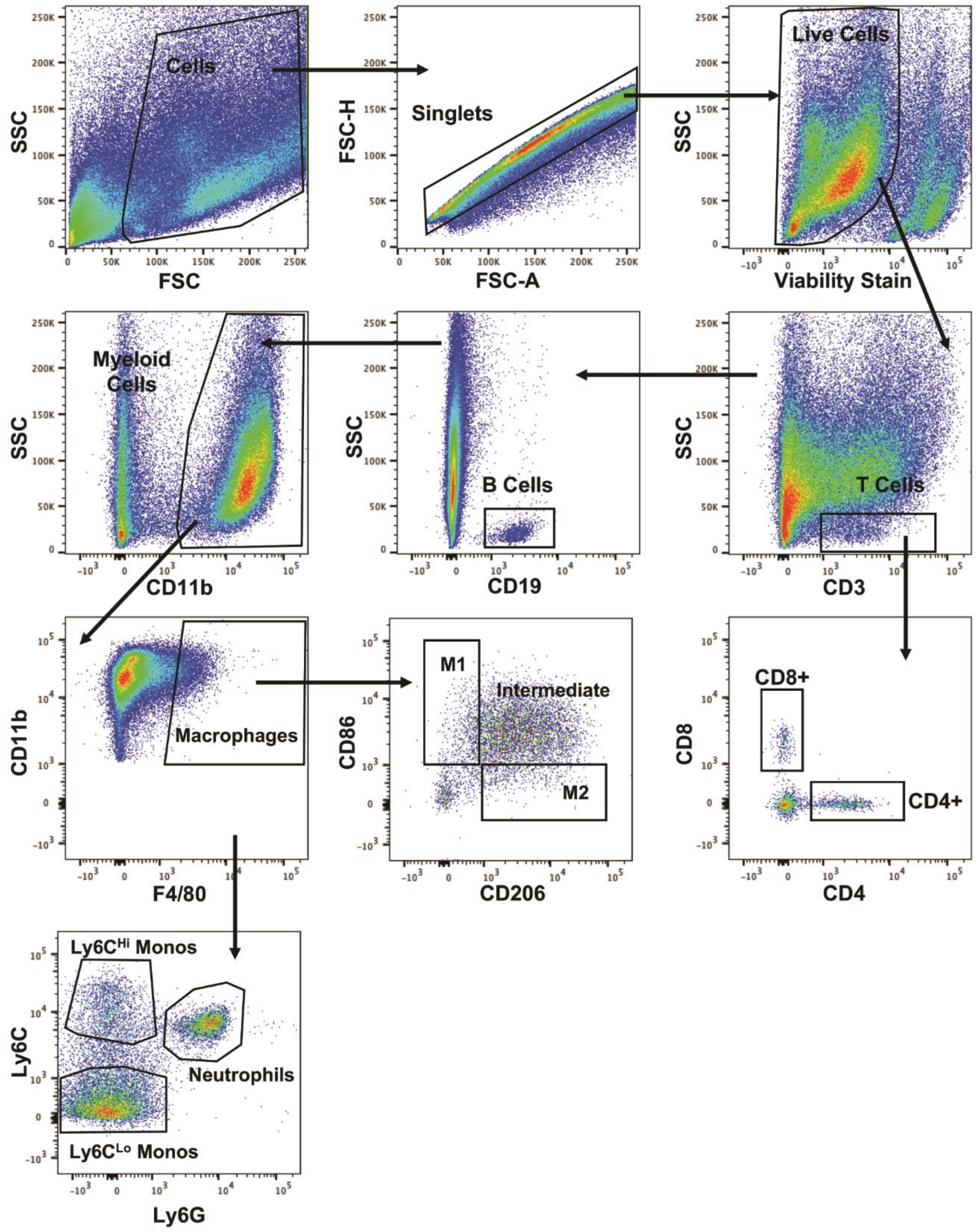

Fig. S6. Gating strategy for inflammatory cell profiling analysis. Single cell suspension of tissue samples were created and stained for subsequent flow cytometry analysis. Single cells were identified and live cells were selected. T cells were identified by selecting as low side scatter CD3 positive events and then sorted into CD8 positive cytotoxic T cells and CD4 positive helper T cells. B cells were low side scatter CD19 positive events. Myeloid cells were identified as being CD11b positive. Macrophages were identified as CD11b and F4/80 positive events then split into CD86 positive M1 and CD206 positive M2 subsets. Neutrophils are identified as Ly6G positive myeloid cells. Monocytes are classified as Ly6G negative myeloid cells and split into Ly6C^{Hi} IM monocytes and Ly6C^{Lo} AM monocytes.

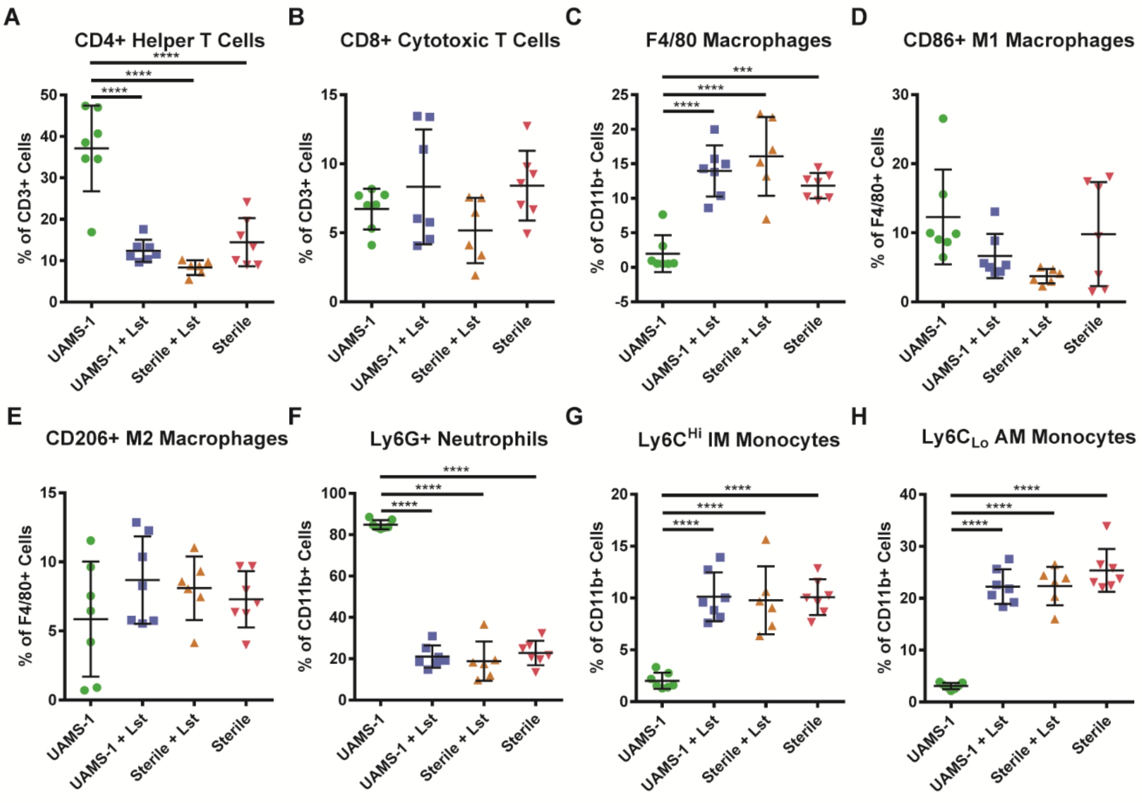

Fig. S7. Percent of parent inflammatory cells at 1 week after implantation of BMP-2–loaded lysostaphin-delivering hydrogels. One week following segmental defect creation and implant placement, mice were euthanized and the implant and surrounding tissue were recovered and flow cytometry was performed to enumerate the total number of inflammatory cells present. (A) CD4+ helper T cells as a percent of CD3+ cells, (B) CD8+ cytotoxic T cells as a percent of CD3+ cells, (C) F480+ macrophages as a percent of CD11b+ cells, (D) CD86+ M1 macrophages as a percent of F480+ cells, (E) CD206+ M2 macrophages as a percent of F4/80+ cells, (F) Ly6G+ neutrophils as a percent of CD11b+ cells, (G) Ly6C^{Lo} AM monocytes as a percent of CD11b+ cells, and (H) Ly6C^{Hi} IM monocytes as a percent of CD11b+ cells were analyzed. Ordinary one-way ANOVA with a Tukey post hoc test, or Kurskall-Wallis with Dunn's test for non-parametric data was used. Means ± SD. N=6-7 per group. ***$P < 0.001$, ****$P < 0.0001$.

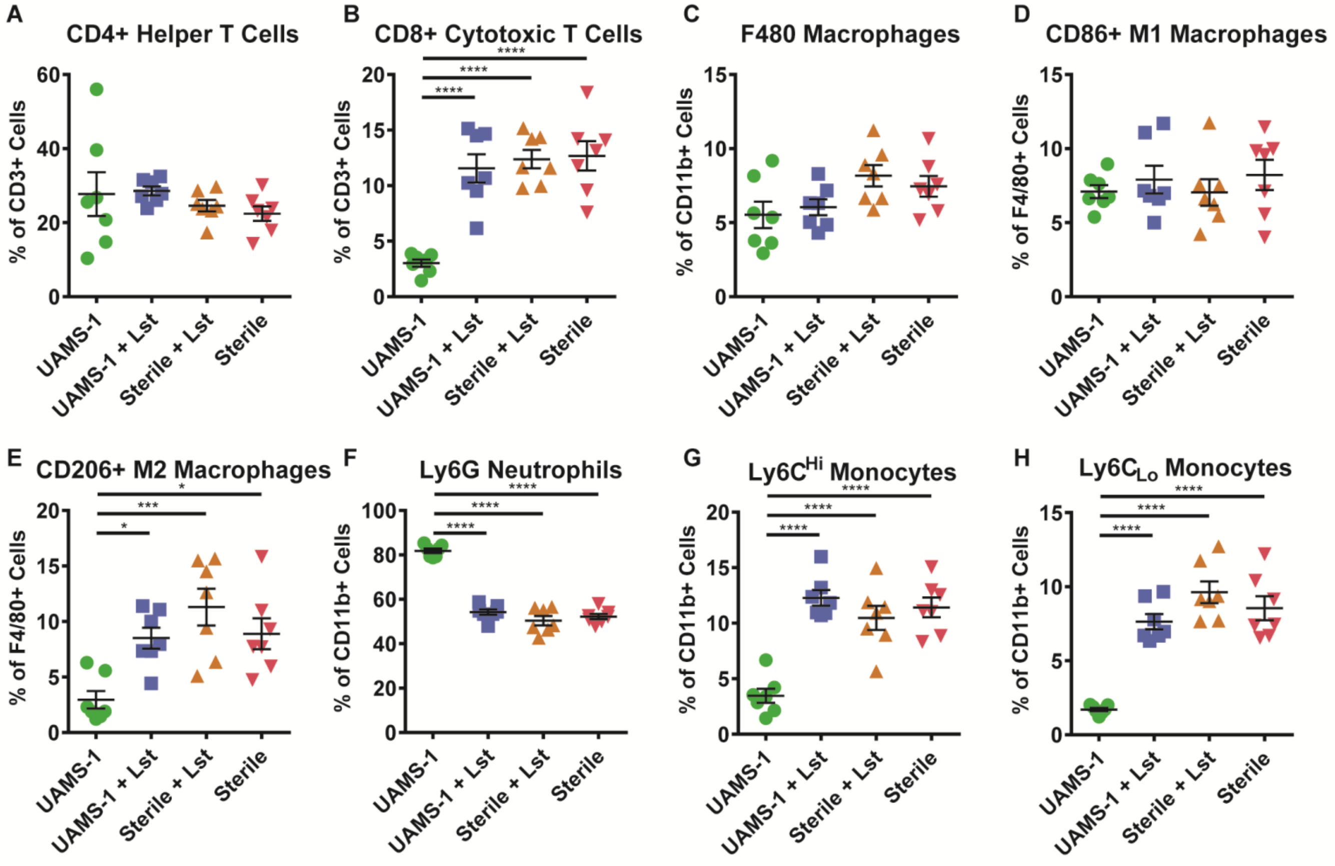

Fig. S8. Percent of parent inflammatory cells at 4 weeks after implantation of BMP-2–loaded lysostaphin-delivering hydrogels. Four weeks following segmental defect creation and implant placement, mice were euthanized and the implant and surrounding tissue were recovered and flow cytometry was performed to enumerate the total number of inflammatory cells present. (A) CD4+ helper T cells as a percent of CD3+ cells, (B) CD8+ cytotoxic T cells as a percent of CD3+ cells, (C) F480+ macrophages as a percent of CD11b+ cells, (D) CD86+ M1 macrophages as a percent of F480+ cells, (E) CD206+ M2 macrophages as a percent of F4/80+ cells, (F) Ly6G+ neutrophils as a percent of CD11b+ cells, (G) Ly6C^{Lo} AM monocytes as a percent of CD11b+ cells, and (H) Ly6C^{Hi} IM monocytes as a percent of CD11b+ cells were analyzed. Ordinary one-way ANOVA with a Tukey post hoc test, or Kurskall-Wallis with Dunn’s test for non-parametric data was used. Means ± SD. N=7 per group. *P<0.05, ***P < 0.001, ****P < 0.0001.

Table S1. Immune cell profiling antibody characteristics. Details regarding flow cytometry antibody targets, clones, fluorophores, test volume used per sample, and BioLegend catalog number.

ANTIBODY	CLONE	FLUOR	TEST VOLUME (µL)	CATALOG #
**CD206**	C068C2	AF488	0.75	141709
**CD19**	6D5	BV421	1.575	115537
**CD4**	GK1.5	BV605	1.5	100451
**CD8**	53-6.7	BV785	1.5	100749
**LY6C**	HK1.4	BV510	1.575	128033
**F480**	BM8	APC	1.575	123115
**CD3**	145-2C11	PE-Cy7	1.575	100319
**LY6G**	1A8	APC-Cy7	1.5	127623
**CD11B**	M1/70	BV711	1.575	101241
**CD86**	GL-1	PE	1.5	105007
**Zombie Red™ Fixable Viability Kit**	n/a	n/a	1	423109
